# Understanding the bacillary load and host interaction to design a point-of-care test to diagnose tuberculosis

**DOI:** 10.3389/ftubr.2023.1243479

**Published:** 2023-08-16

**Authors:** Pere-Joan Cardona

**Affiliations:** ^1^Northern Metropolitan Clinical Laboratory, Microbiology Department, Hospital Universitari “Germans Trias i Pujol”, Catalonia, Spain; ^2^Experimental Tuberculosis Unit, Germans Trias i Pujol Research Institute (IGTP), Catalonia, Spain; ^3^Centro de Investigación Biomédica en Red de Enfermedades Respiratorias (CIBERES), Madrid, Spain; ^4^Genetics and Microbiology Department, Autonomous University of Barcelona, Cerdanyola del Vallès, Spain

**Keywords:** *Mycobacterium tuberculosis*, TB spectrum, dynamic hypothesis, bubble model, diagnosis, cortisol, poverty, point-of-care

## Abstract

Designing of a Point-of-care test to diagnose tuberculosis (TB) is not an easy task. This viewpoint stems from the dichotomous diagnostic approach, based on the bacillary load estimated in latent tuberculosis infection (LTBI), thanks to the isoniazid chemoprophylaxis strategy, as well as the importance of imaging to differentiate between LTBI and TB. It integrates the “TB spectrum” elucidated through positron emission tomography-computed tomography scan (PET-CT) to highlight the dynamic nature of TB lesions. Additionally, it emphasizes the relevance of animal models that support this perspective, including the drainage of bacilli through foamy macrophages, which aids in understanding LTBI and its chemoprophylaxis, and the significance of lung anatomy in TB induction. Especially the role of interlobular septa and the encapsulation process and its role in lung lobe predilection impact disease progression. Moreover, it acknowledges the gender bias in TB, as its incidence is significantly higher in men across various socioeconomic circumstances, suggesting an unidentified biological mechanism. For a comprehensive approach, the impact of stress and cortisol levels is suggested as a new parameter to be considered, given their association with poverty, and social inequity, and their tendency to be higher in men. All this information has to be contemplated when designing an accurate point-of-care test. The test should encompass the complexity of TB and necessarily integrate both bacillary and host response parameters. It also should cover the diagnosis of extrapulmonary TB, and pay attention to immunosuppressed and pediatric population.

## Introduction

There is an urgent need to find novel rapid tests for the diagnosis of tuberculosis (TB), which are inexpensive and easy to perform in challenging environments. This is crucial in order to achieve the EndingTB-2030 objective (1). Currently, one-third of TB patients go undiagnosed, and this situation is even worse in the African region, where the percentage rises to 50%. Therefore, point-of-care diagnosis of TB poses a significant challenge for the scientific community.

Discovering such methodologies necessitates a thorough understanding and a continuous reconsideration of the relationship between *Mycobacterium tuberculosis* load and its natural history of infection.

## The origin of *Mycobacterium tuberculosis*

Tuberculosis (TB) remains a significant cause of mortality worldwide. It belongs to the genera *Mycobacterium*, which emerged relatively recently, approximately 36 million years ago (2), as an evolution of the *Nocardia* genera. This bacteria began developing a robust outer membrane to protect the peptidoglycan layer using large fatty acids known as mycolic acids, which can reach chain lengths of up to 60 carbon atoms (3). The most common ancestor of *Mycobacterium* closely resembles the *M. abscessus-chelonae* complex (4), a rapid growing mycobacteria (RGM), incorporating even larger mycolic acids (up to 90 carbon atoms), which enhance resistance to environmental conditions, and gives the characteristic acid-fast positivity of this genera (3). By abandoning the production of hyphae observed in *Nocardia, Mycobacterium* were able to colonize a new ecological niche: soil amoebae. Within these amoebae, mycobacteria can survive intracellularly and benefit from additional protection against harsh environmental conditions present in the soil, particularly during the cystic phase of the amoebae's life cycle (5). Amoebae play a crucial role in regulating bacterial populations in the soil and maintaining a balanced rhizosphere microbiome, which provides essential minerals, modulates plant hormonal balance, and suppresses potential pathogens (6).

Evolution of RGM to slow growing mycobacteria (SGM) is closely tied to their enhanced ability to enter amoebae, which involves adaptation of the *mce* operon. This adaptation allowed for better specialization within this niche, leading to the gradual loss of gens required for essential nutrient uptake and metabolism in the external environment, which are no longer necessary (7). This specialization extends beyond amoebae to their evolutionary counterparts found in multicellular organisms, specifically macrophages (8). Consequently, SGM can be transmitted to fish and amphibians through water, especially after acquiring a new virulent mechanism (ESAT-6) that allowed the capacity to avoid the phagolysosome fusion (9), allowing a better transmission among macrophages. This is the case of *M. marinum*, and also through aerosols to mammals and our non-human primate ancestors, as seen in the case of *M. kansasii*. The modification of the outer membrane's polarity plays a crucial role in this process. The transition from hydrophilic lipooligosaccharides and phenolic glycolipids to hydrophobic phthiocerol dimycocerosates, di- and pentaacyl trehaloses and sulfoglycolipids which confer increased hydrophobicity, has facilitated more efficient aerosol transmission (10). This transition occurred approximately 3 million years ago, leading to the emergence of the most recent common ancestor of the *M. tuberculosis* complex, resembling a *Canetti*-like strain (11), becoming the transit toward obligate parasites of humans.

## Direct detection of *Mycobacterium tuberculosis*: finding the bacilli at any prize?

The detection of acid-fast bacilli has been a key method for diagnosing *M. tuberculosis* (Mtb), particularly considering that the primary route of infection is through the respiratory system. Before the advent of molecular biology tools, the acid-fast stain of sputum was the primary diagnostic tool for tuberculosis (TB). The presence of even a single bacillus in the sputum is indicative of TB. This technique has taught us several important lessons.

Firstly, the acid-fast stain has limited sensitivity. It requires a concentration of 5,000–10,000 bacilli per milliliter (mL) of sample for detection (12). A positive smear indicates a significant burden of bacilli and suggests a severe form of the disease (13). This underscores the importance of active and intensive contact surveillance for individuals who have been in close contact with TB patients.

The sensitivity of detection is increased when the sample is cultured after decontamination. In this case, only 10 bacilli/mL of sputum are required for a positive result. However, culturing requires specialized infrastructure that may not be available in many local laboratories, especially in countries with a high incidence of TB. Consequently, significant efforts have been made to incorporate molecular biology methodology in various settings, as they do not require specific infrastructure. Notwithstanding, molecular methods have lower sensitivity compared to culturing, with a lower threshold of 15.6 bacilli/mL (14).

## Dichotomous vs. spectrum model of TB diagnosis management

In essence, the diagnosis of TB still follows a dichotomous approach based on the sensitivity of culturing, which requires a minimum of 10 bacilli per milliliter (mL) of sputum, ideally tested using three consecutive morning sputum samples. However, let's imagine if we had a more sensitive technique that could detect 1 bacillus per mL. Would that be sufficient to validate a TB diagnosis?

If we calculate that the lungs drain approximately 500 mL of alveolar fluid every day, it can be argued that in a TB case, at least 5,000 bacilli are drained from the lesions daily. Refining the calculations, considering that TB predominantly affects the upper lobes (which represent 20% of the total pulmonary volume), the fluid volume is reduced to 100 mL, or even 50 mL if we focus on a single upper lobe. This means that at least 500 bacilli need to be drained from the infected area during sleep (morning sputum are the best) to be detectable through culturing and confirm a TB case.

**Figure 1:**
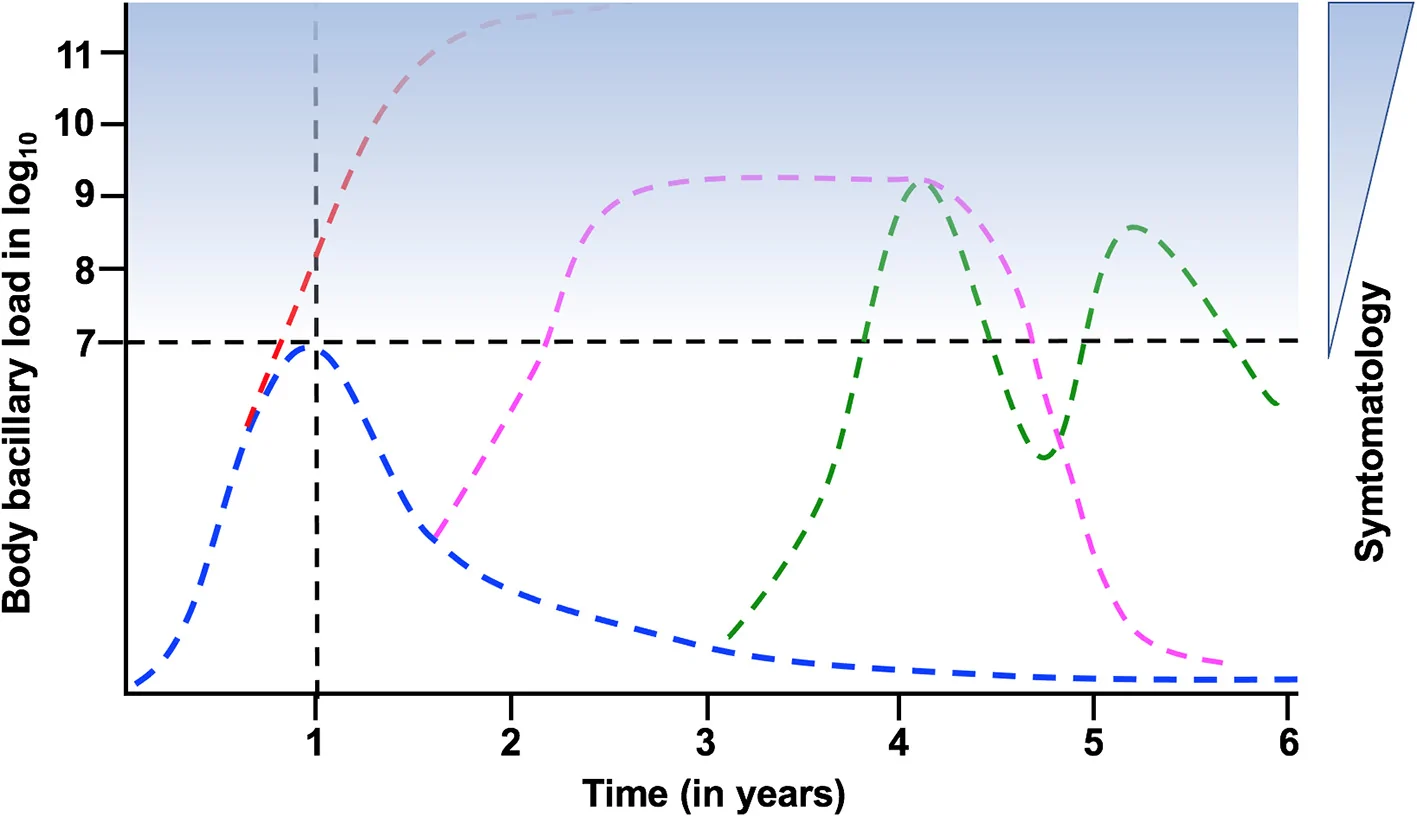
Evolution of the bacillary load after the acquisition of latent tuberculosis infection (LTBI). Doted lines mark the “LTBI threshold” in terms of bacillary load, and the 1^st^ year after infection, when the possibility of developing TB is higher. Blue stain range indicates the possibility of symptomatology. In blue the kinetics of LTBI; in red the progression toward active TB during the 1^st^ year; in pink acquisition of TB and recovery; in green the possibility of cyclic bacillary regrowth and control.

This calculation of bacillary load aligns with the dichotomous model of TB, which originated with the initiation of preventive TB therapy by the US Public Health Service in the 1950s (15). Treatment with isoniazid (INH) for 9 months results in a reduction of TB cases by approximately 90% in close contacts of active TB patients (16). This effect is particularly significant during the first year after infection, when the risk of developing TB is higher, especially in children with < 5 year of age and immunosuppressed people (17, 18), to become negligible around 8 years post-infection (19). Considering that spontaneous mutation against INH occurs once per 6.5–7 log_10_ bacilli (20), and that no INH resistance has been described after chemoprophylaxis, it can be inferred that individuals recently infected, with latent tuberculosis infection (LTBI), have fewer than 6.5–7 log_10_ bacilli in their body (Figure 1). Upward this threshold, the presence of TB is considered, requiring treatment with at least three drugs to avoid the selection of strains with spontaneous mutations, which could lead to a “fall and raise” phenomenon (21).

In general, the dichotomous approach is used in contact tracing, where LTBI is defined in individuals with a positive Mantoux test or interferon gamma release assay (IGRA) but no chest X-ray lesions, while pulmonary TB is diagnosed when a lesion is detected and confirmed by microbiological methods. This pragmatic approach links the size of the lesion with the bacillary load and has been one of the key elements in TB diagnosis. However, this paradigm may change with the increasing sensitivity of imaging techniques, such as chest computed tomography (CT), which can identify micronodules that are impossible to detect on chest X-rays (22). Regardless of the feasibility of the method, the question remains whether it is worthwhile to change the existing paradigm.

Some years ago, with the onset of PET-TAC, a concept emerged that challenged the dichotomous model of TB diagnosis: the “TB spectrum” (23, 24). The TB Spectrum suggests that the evolution from LTBI to TB involves intermediate steps, questioning the notion of an “LTBI threshold”. It suggests that there can be cyclic episodes of bacillary regrowth and control, as well as natural healing of the lesions (13). Not to mention exogenous reinfections, of special interest in countries with a high TB incidence (25). Furthermore, the question of infection in naïve subjects arises, whether it results from a single bacilli infection or multiple consecutive infections (26).

The TB spectrum approach also emphasizes the relevance of symptomatology in the diagnostic algorithm. However, TB symptoms can be variable among patients and often indeterminate. The common guideline that suggests “2 weeks of cough may indicate TB” is vague, and physicians must consider various social, economic, and geographic factors to make an accurate assessment. Consequently, there is a significant diagnostic delay, with a median of 52 days and up to 6.5 months in disseminated TB (27). It is worth nothing that TB does not necessarily have to be linked to symptomatology or organ failure. In fact, the low severity and asymptomatic nature of TB in the Paleolithic era may explain the origin of Mtb (28).

Considering the obligate nature of Mtb infection in humans, it seems reasonable to orientate an eradication policy toward the detection of Mtb at all cost, in a massive and cost-effective manner. This approach is achievable when the bacillary load is detectable in respiratory samples. However, the challenge lies in the detection of extrapulmonary TB, where the bacillary load may be lower or more difficult to access.

### The host-pathogen interface: on foamy macrophages, interlobular septa and soap bubbles

**Figure 2:**
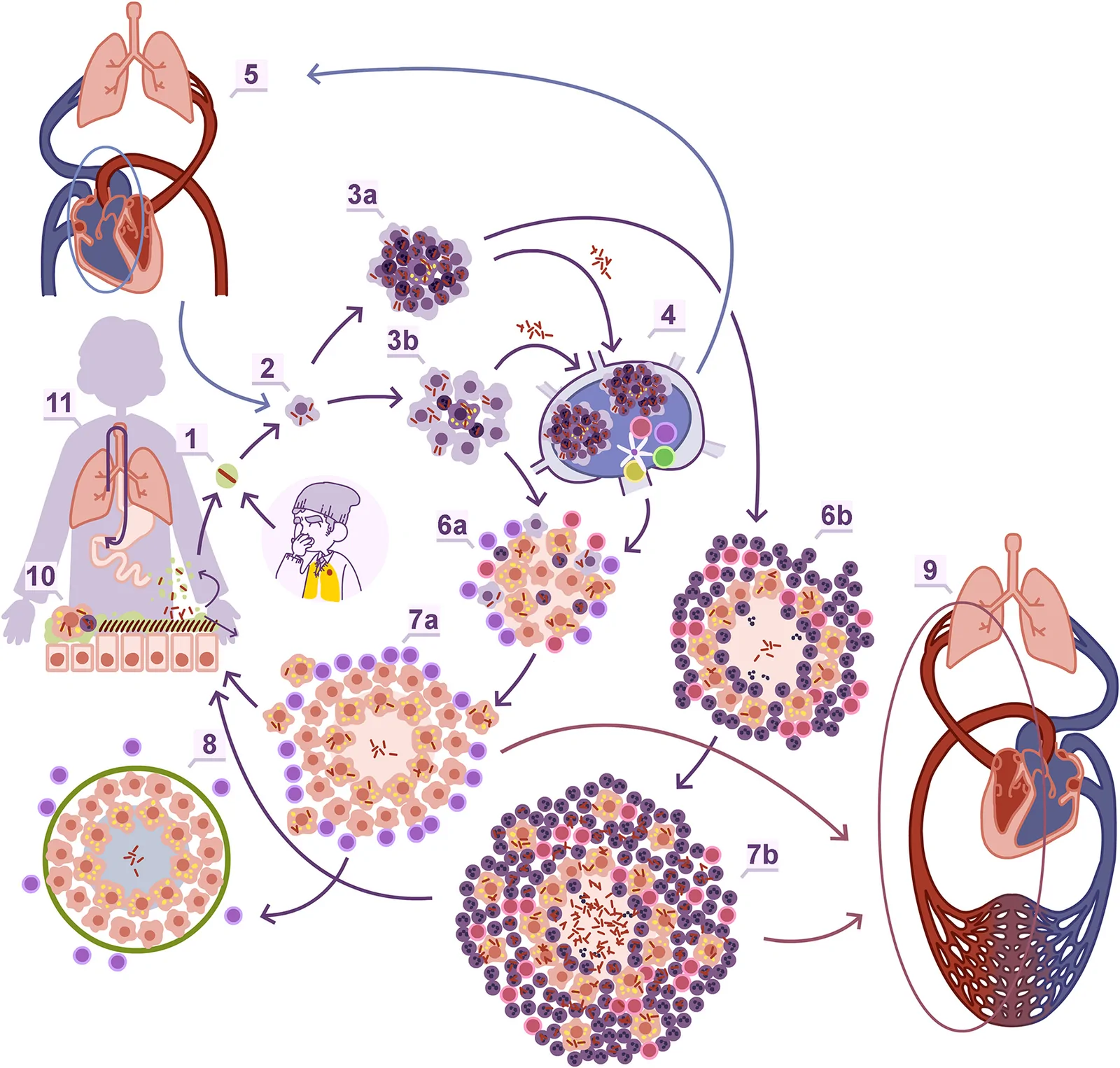
Natural history of Mtb infection. Entrance to the alveoli through aerosol (1). Macrophage entrance (2). The onset of the granuloma, which depending on the infiltration percentage of polymorphonuclear leukocytes (PMNs) can be identified as proliferative (3b) or exudative (3a) when it is low or high, respectively. The interaction with the regional lymph node (4), where Mtb can also infect macrophages, generate lesions which can be a source of new reinfection of the lung once infected macrophages or extracellular bacilli reach the inferior vena cava to the right auricula and are pumped again through the pulmonary artery. Once the adaptive immunity is triggered there is a control in the bacillary load in the case of the proliferative granulomas thanks to a predominant Th1 response (6a, 7a) or a progressive increase thanks to the enhanced entrance of PMNs through a Th17 biased response, that allows an extracellular growth of the bacilli (6b, 7b) and the angiogenesis of new more fragile capillary, than can easily break and promote extrapulmonary dissemination through the pulmonary veins (9). Proliferative lesions are rapidly encapsulated (8). In both lesions there is a constant drainage of dormant bacilli carried by foamy macrophages toward the bronchial tree (11) which are mainly drained toward the gastrointestinal tract (12) but can re-enter the lung parenchyma again after incorporating in the aerosols produced in the bronchioles (1). Obtained with permission from Cardona et al. (86).

Experimental modeling in laboratory animals, particularly mice, has played a crucial role in understanding the natural history of Mtb infection (Figure 2). These models have been instrumental in demonstrating the critical role of interferon-gamma (IFN-g) and Th1 response in activating infected alveolar macrophages and controlling the infection (29). They have also highlighted the significance of foamy macrophages (FM), which are old activated macrophages, in carrying dormant bacilli out of the granuloma and draining them through the bronchioles toward the upper bronchi (30, 31). The periodic collapse of bronchioles during respiration generates aerosols (32), providing a pathway for endogenous reinfection after the lysis of infected FM and incorporation of dormant Mtb into these aerosols. This mechanism supports the “dynamic hypothesis” (33), which explains several aspects of TB development. For example, it elucidates why the incidence of TB decreases significantly 1 year after infection due to the constant drainage of bacilli through the gastrointestinal tract, reducing the chances of reinfection over time. It also clarifies the rationale behind administering INH for LTBI, as INH has no effect on dormant bacilli. INH chemoprophylaxis is useful when given for an extended period, as it corresponds to the average bacillary “clean up” time from the granulomas through the gastrointestinal tract. Maintaining constant levels of INH helps prevent the regrowth of those dormant bacilli endogenously reintroduced to the lung.

The Mtb infection in minipigs provided another clue. Is not solely about the immune response, but there is also a mechanical component in the lung that needs to be considered. The interlobular septa, which divide de entire pulmonary parenchyma into smaller sections to facilitate respiration through the contraction of the diaphragm, play a role in this regard (34). These septa contain fibroblasts that can detect any injury in the parenchyma, leading to mechanical stresses on the tissue. This triggers a switch to the myofibroblast phenotype (35), characterized by elongation, stress fiber production, and rapid encapsulation of the granulomas within approximately 10 days (36) (Figure 2).

Infection of the C3HeB/FeJ mouse strain has helped us understand how to overcome this encapsulation process. This strain exhibits exponentially increase in bacillary load due to the neutrophilic infiltration surrounding infected FM, leading to the generation of neutrophil extracellular traps, that facilitate the extracellular growth of Mtb (37) and the development of cording, which further amplifies inflammation (38). The drained and infected FM also play a role by generating daughter lesions around the initial one, which are rapidly infiltrated by polymorphonuclear leukocytes (PMNs) and give rise to subsequent generations of daughter lesions. Eventually, all these lesions grow and merge, resulting in massive irregular necrosis that undergoes liquefaction (39). This process resembles the formation of soap bubbles and has been simulated using an “*in silico*” model known as the “bubble model” (40). The significance of daughter lesions in the progression toward liquefacted lesions has been confirmed in experimental macaque models (41), and it aligns with recent studies using human samples (42). The enlargement of the lesions and modification of the parenchyma also requires neovascularization, resulting in the formation of a fragile capillary network that can be easily rupture and disseminate bacilli into the pulmonary veins, leading to systemic dissemination (43) (Figure 2). From a point of view focused on POC strategies, the use of a CT scan is not a viable alternative, but the detection of increased angiogenesis or collagen turnover (e.g., metalloproteinase activity) could support a biosignature approach.

### Why upper lobes and men?

Pulmonary TB in immunocompetent individuals tends to primarily affect the upper lobes of the lungs. In fact, bats (that hangs upside down during most part of the day) develop TB mostly in the bases of their lungs. Accordingly, in four-legged animals the lesions concentrate in the dorsal regions of the lung (44, 45). There are several factors that contribute to this localization. Firstly, the small breathing amplitude in the upper lobes creates an environment that facilitates the local accumulation of bacilli, making it more favorable for disease to occur (46). Additionally, the upper lobes have larger alveoli, which experience increased strain due to the effect of gravity (47). This increased strain can have an impact on the reactivity of fibroblasts in the upper lobes, potentially making them less responsive.

The combination of these factors creates a stressful environment in the upper lobes of the lungs, leading to the attraction of PMNs. The presence of PMNs promotes the extracellular growth of the bacilli and enables them to overcome the encapsulation process that typically occurs in response to infection. The reduced reactivity of fibroblasts in the upper lobes may also contribute to the diminished encapsulation efficacy. These mechanisms collectively contribute to the predilection of pulmonary TB for the upper lobes in immunocompetent individuals.

The higher incidence of TB in men compared to women has been observed globally, with a ratio of approximately 65:35 in WHO reports (48). Several factors contribute to this gender disparity. One significant factor is the under-notification of TB cases in women due to inequities in accessing healthcare systems, particularly in developing countries (49). However, this gender imbalance persists worldwide, suggesting the presence of biological sex-biases that are not yet fully understood. These biases may be related to factors such as sexual hormones, sex-related genetic backgrounds and genetic regulations, metabolism, or other yet-to-be-identified factors (50). Further research is needed to elucidate the specific mechanisms underlying the higher susceptibility of men to TB.

One potential explanation for the development of severe TB disease in humans relates to the neuroendocrine stress response. This response, known as the “fight or flight” reaction, involves the secretion of glucocorticoids (GCs), such as cortisol (51). Research has shown that lower socioeconomic status is associated with chronic stress and elevated cortisol levels (52). The onset of the agricultural revolution introduced social imbalances and transitioned society into an unequal one, diverging from the collaborative nature of the Paleolithic era (53, 54).

The development of severe forms of TB can be attributed to chronic cortisol elevation rather than an increase in Mtb virulence. In fact, modern Mtb lineages are generally less proinflammatory (55) and less likely to induce severe lesions. This perspective aligns with the concept of TB as a disease affecting lower social classes and reflecting societal inequality (56). Several observations support this hypothesis. For example, studies have documented elevated basal cortisol levels in farmers in Kenya who rely solely on agriculture for their income (57), and in non-politically influential men of the Bolivian Tsimane forager-horticulturalists (58). Additionally, studies in various primate species have demonstrated that subordinate individuals exhibit higher resting cortisol levels compared to their dominant counterparts (59). Even in the absence of baseline differences, research has found that men produce higher circulating cortisol levels than women during psychological stress tests (60–62). This may explain why major depression increases the risk of TB, but specifically in men, as observed in a nationwide population study in Korea (63).

In immunosuppressed individuals, such as children under the age of 5 and AIDS patients, the tropism for the upper lobes in pulmonary TB is not necessarily observed. In these cases, the development of TB is associated with inadequate cellular immunity. The lack of activation of infected macrophages contributes to easier dissemination of the bacilli, as there is limited activation of infected macrophages due to reduced Th1 response, as well as reduced attraction of PMNs caused by a diminished Th17 response (Figure 2). Consequently, TB lesions can develop in all pulmonary lobes in these hosts, and cavitation of the lesions is less frequent. Additionally, systemic dissemination of the infection is more common, leading to the development of extrapulmonary lesions with greater frequency (64).

## Discussion on existing methodologies

This perspective highlights several aspects that need to be considered when designing a new POC test for diagnosing tuberculosis (TB).

On the direct approach side, there are various methods for detecting the presence of Mtb. Culture systems, particularly those using liquid media such as the Mycobacteria Growth Indicator Tube (MGIT) has the highest sensitivity. Smear detection using light-emitting diode (LED) based fluorescence microscopy has also been improved (65). Antigenic methods have focused on detecting lipoarabinomannan (LAM), a major component of the Mtb cell wall. Soluble LAM, actively secreted by Mtb and infected macrophages, can be detected in urine and holds potential as a POC test (66). However, the most widely used method with high sensitivity is the Xpert MTB/RIF test, which utilizes DNA detection for Mtb and rifampicin resistance in various specimen types (67–70).

On the indirect approach side, the host interface is considered. The classical test uses purified protein derivative (PPD) of a Mtb extract, or tuberculin, standardized by Florence Seibert (71). Its intradermal injection using the Mantoux technique induces a delayed-type hypersensitivity response in individuals with a LTBI. A more specific version uses antigens such as ESAT-6 and CFP-10 to avoid cross-reactivity with BCG-vaccinated or most of non-TB mycobacteria infected individuals (72, 73). On the other hand, Interferon-gamma release assays (IGRAs) detect specific Th1 effector memory cells in the blood by quantifying IFN-g production upon stimulation with antigens like ESAT-6 and CFP-10 using ELISA or ELISPOT technologies (74).

Direct methodologies are valuable for diagnosing TB, while indirect methods cannot distinguish between LTBI and TB. Further refinement may involve using easily obtained samples like saliva for Mtb DNA detection or detecting host salivary proteins as biosignatures, although specificity remains a challenge (75, 76). Biosignatures based on metabolomics, IFN-g induced agents, or microRNAs have also been explored (74, 77), also in blood-based tests, including dry blood testing (78, 79).

The main challenge with these approaches is that the cellular immune response cannot determine the localization of the lesion or the risk of progression to TB. In contrast, the humoral response offers a combination of bacillary load detection and host response. Antibody production is primarily induced after extracellular bacillary growth, making it a potential marker for TB diagnosis. This methodology has been under development for several years (80), and has shown promising results, particularly when combined with IGRAs (81), an approach that merits further development. However, due to the proliferation of inaccurate tests, the Indian Ministry of Health banned the import and sale of antibody-based TB tests in 2012 (82, 83).

Special attention should be given to TB diagnosis in children (84), as they often present with atypical pulmonary TB and face challenges in obtaining good sputum samples. Additionally, there is an increased risk of extrapulmonary dissemination. Detecting specific metabolomic biosignatures in urine shows promise as a potential option for diagnosing TB in children, although further development is needed in terms of cost and feasibility (85).

In conclusion, tuberculosis (TB) diagnosis remains a significant global healthcare challenge. Current diagnostic methods have limitations in sensitivity and infrastructure requirements, hindering timely and accurate detection, especially in resource-limited settings and for extrapulmonary cases. A paradigm shift is needed, moving toward a comprehensive understanding of TB as a spectrum of infection, from latent to active disease. By considering the dynamic nature of TB and key factors such as foamy macrophages, interlobular septa, and anatomical location, innovative diagnostic approaches can be developed. Factors like male susceptibility and the impact of chronic stress emphasize the need to consider social, genetic, and environmental aspects in TB diagnosis and management. Collaboration and innovation are crucial to develop rapid, sensitive, and cost-effective diagnostic tests that can be implemented in challenging environments. By improving early detection and reducing diagnostic delays, we can effectively control the spread of TB and work toward the goal of ending the disease by 2030.

## Data availability statement

The original contributions presented in the study are included in the article/supplementary material, further inquiries can be directed to the corresponding author.

## Author contributions

P-JC has conceptualized and written the article.
